# Community health worker programmes after the 2013–2016 Ebola outbreak

**DOI:** 10.2471/BLT.15.164020

**Published:** 2016-06-02

**Authors:** Henry B Perry, Ranu S Dhillon, Anne Liu, Ketan Chitnis, Rajesh Panjabi, Daniel Palazuelos, Alain K Koffi, Joseph N Kandeh, Mamady Camara, Robert Camara, Tolbert Nyenswah

**Affiliations:** a Department of International Health, Johns Hopkins Bloomberg School of Public Health, Baltimore, MD, United States of America (USA).; b Division of Global Health Equity, Brigham and Women's Hospital and Harvard Medical School, Boston, USA.; c Earth Institute, Columbia University, New York, USA.; d Communication for Development Section, United Nations Children's Fund, New York, USA.; e Last Mile Health, Boston, USA.; f Partners In Health, Boston, USA.; g Directorate of Primary Health Care, Ministry of Health and Sanitation, Freetown, Sierra Leone.; h Earth Institute, Columbia University, Conakry, Guinea.; i Directorate of Prevention and Community Health, Ministry of Health and Hygiene, Conakry, Guinea.; j Liberia Incident Management System for Ebola Response, Ministry of Health and Social Welfare, Monrovia, Liberia.

The 2013–2016 Ebola virus disease outbreak in West Africa exposed an urgent need to strengthen health surveillance and health systems in low-income countries, not only to improve the health of populations served by these health systems but also to promote global health security.^1^ Chronically fragile and under-resourced health systems^2^ enabled the initial outbreak in Guinea to spiral into an epidemic of over 28 616 cases and 11 310 deaths (as of 5 May 2016)^3^ in Guinea, Liberia and Sierra Leone, requiring an unprecedented global response that is still ongoing. Control efforts were hindered by gaps in the formal health system and by resistance from the community, fuelled by fear and poor communication. Lessons learnt from this Ebola outbreak have raised the question of how the affected countries, and other low-income countries with similarly weak health systems, can build stronger health systems and surveillance mechanisms to prevent future outbreaks from escalating.^4^ Factors that were important in the growth and persistence of the Ebola virus outbreak were lack of trust in the health system at the community level, the spread of misinformation, deeply embedded cultural practices conducive to transmission (e.g. burial customs), inadequate reporting of health events and the public’s lack of access to health services.^1^ Community health workers are in a unique position to mitigate these factors through surveillance for danger signs and mobilization of communities when an outbreak has been identified. In this paper we make the case for investing in robust national community health worker programmes as one of the strategies for improving global health security, for preventing future catastrophic infectious disease outbreaks and for strengthening health systems.

Community health workers provide health education, gather information and deliver basic curative and preventive services at the community and household levels. They were first deployed in China nearly a century ago and have been deployed by both nongovernmental organizations (NGOs) and national governments over the past half-century.^5^ Although community health workers play diverse roles, they share common features: they receive limited formal training and are recruited from and work in their own communities.^6^ They are therefore uniquely positioned to promote healthy household practices and appropriate health-care-seeking behaviours. Large-scale national community health worker programmes are the cornerstones of primary health-care systems in many countries that have been pacesetters in improving the health of their populations, such as Brazil, Ethiopia, Malawi, Nepal and Rwanda.^7^ Yet the failure of several national community health worker programmes in the 1970s and 1980s resulted in a loss of momentum for the movement. As a result of the growing success of the current programmes,^5^^,^^7^ there is renewed global interest in using community health workers to strengthen primary health-care systems towards the goals of achieving universal health coverage and ending preventable child and maternal deaths.

During the most recent Ebola outbreak, community health workers played several important roles. They worked with community leaders, going house to house to provide important information about Ebola and searching for active cases and contacts,^8^ and they helped local religious leaders to expand their education and outreach strategies, especially in efforts to reduce transmission during funerals and burials. Many community-based agents, including community health workers working with NGOs, were deployed for contact tracing, community sensitization, promotion of epidemiologically and culturally appropriate protective practices, and data collection.^8^ Networks of community health workers played key roles in limiting the spread of Ebola virus infection within Nigeria in July 2014.^9^ Community health workers who were normally engaged in polio eradication initiatives were rapidly redeployed to detect patients with Ebola virus and trace their contacts. Activating an emergency response centre and using all available public health assets enabled Nigeria to quickly end its 2014 Ebola virus outbreak,^9^ which, due to the much larger urban populations, could have been a far worse global health crisis than elsewhere in West Africa.^10^ Community health workers and local networks of organized social mobilizers played similar roles in the elimination of polio transmission in Uttar Pradesh, India, in 2011.^11^

In the light of the Ebola virus crisis three key developments needed for resilient health systems have been identified: (i) improved disease surveillance; (ii) greater trust and engagement with communities; and (iii) a stronger health workforce.^4^ Community health workers can make important contributions to each of these areas. First, they can help limit the spread of future outbreaks of diseases of public health importance by early detection of incident cases and rapid containment of these. The Ebola virus outbreak brought the importance of, and current gaps in, disease surveillance systems to the forefront of the discussion on health systems and global health security.^1^ Patients’ use of health care decreases exponentially with distance and therefore surveillance strategies based exclusively on facility-based detection will miss outbreaks that begin in communities living far away from a health facility.^12^^,^^13^ This was evident during the current outbreak, as the first Ebola virus cases occurred in secluded villages in south-eastern Guinea and three months passed before it was recognized that an epidemic had emerged. A similar scenario occurred when the virus first crossed into Sierra Leone and spread intensely in a series of border villages while international response teams presumed the outbreak in Guinea was coming to an end.^14^ Community health workers are therefore particularly important in remote villages or hard-to-reach populations, where people often live hours or even days of travel away from the nearest clinic. The identification of suspected cases by a community health worker can serve as an early warning system for disease outbreaks.

Second, community health workers can play an important role in controlling outbreaks by engaging and educating communities. There is increasing recognition that rapid containment of the spread of Ebola virus was seen in those communities where mobilization efforts resulted in building trust between the community and those who were working to control it.^15^^,^^16^ As demonstrated on a national scale in Ethiopia,^7^ if community health workers have appropriate training, supervision and support, they can play a key role in community-based public health programmes. They can take on the roles of health educators and community organizers, link with existing local social networks to create bonds of trust between communities and health systems, and use existing community resources to encourage community members to access the nearest health services when needed. Community health workers can also make important contributions to the prevention and rapid containment of other infectious diseases through outreach, education and community engagement strategies. The ability to respond quickly in case of an initial outbreak is likely to reduce the risk of widespread infection.

Third, deploying community health workers will contribute to the strengthening and rebuilding of health systems in Ebola-virus-affected countries and beyond. A review of their role in low-, middle- and high-income countries concluded that community health workers can safely and effectively deliver interventions that are low-cost but high-impact including: community case management of serious childhood illness (pneumonia, diarrhoea, malaria and severe acute malnutrition); promotion of good nutrition; and the provision of family planning services.^5^ There is mounting evidence that community health workers can improve the public’s health-seeking behaviours, reduce maternal, neonatal and child mortality, and contribute to the control of human immunodeficiency virus (HIV) transmission and infection, tuberculosis and malaria.^5^

Ethiopia provides the best example in Africa of the success of scaling up, over a 10-year period, of a programme comprising a dual cadre of community health workers: health extension workers (who are professionalized with one year of formal training, paid and serving 2500 people) and health development army volunteers (trained informally by health extension workers and serving 5 to 10 families).^7^ Its programme reaches 92 million people, four times the combined populations of Guinea, Liberia and Sierra Leone. Ethiopia’s community health workers visit all households on a regular basis and have made important contributions to the marked improvements in the control of HIV transmission, tuberculosis and malaria, to the reduction in maternal and child mortality, and to the increase in the prevalence of contraceptive use.^7^

To be effective, large-scale community health worker programmes require long-term, high-level political and financial support, as well as high-quality technical support for planning, management, training, supervision and logistics. Unfortunately, these features were often lacking in earlier community health worker programmes. This led to the collapse of most of them and concern in many quarters today about whether today’s programmes will eventually suffer a similar fate.^5^ Broader health systems strengthening is needed; robust community health worker programmes cannot be scaled up without a functioning health system. However, strengthening the health system without also developing and strengthening the role of community health workers would be a missed opportunity.

Attitudes are changing and the potential benefits of expanded investments in community health worker programmes are more widely recognized today.^17^ There is growing evidence of the effectiveness of community health workers^7^ when they are well trained and well supported.^5^ Strong community health worker programmes can be a key resource not only for global health security but also for accelerating progress towards universal health coverage, ending preventable child and maternal deaths, and eventually achieving health for all.
